# Kinect-Assisted Performance-Sensitive Upper Limb Exercise Platform for Post-stroke Survivors

**DOI:** 10.3389/fnins.2019.00228

**Published:** 2019-03-22

**Authors:** Adyasha Dash, Anand Yadav, Anand Chauhan, Uttama Lahiri

**Affiliations:** Department of Electrical Engineering, Indian Institute of Technology Gandhinagar, Gandhinagar, India

**Keywords:** computer-based-task, stroke, adaptive design, physiology, upper limb

## Abstract

One's ability to use upper limbs is critical for performing activities of daily living necessary for enjoying quality community life. However, after stroke, such abilities becomes adversely affected and it often deprives one of their capability to perform tasks that need coordinated movement in the upper limbs. To address issues with upper limb dysfunction, patients typically undergo rehabilitative exercises. Given the high patient to doctor ratio particularly in developing countries like India, conventional rehabilitation with patients undergoing exercises under one-on-one therapist's supervision often becomes a challenge. Thus, investigators are exploring technology such as computer-based platforms coupled with cameras that can alleviate the need for the continuous presence of a therapist and can offer a powerful complementary tool in the hands of the clinicians. Such marker-based imaging systems used for rehabilitation can offer real-time processing and high accuracy of data. However, these systems often require dedicated lab space and high set-up time. Often this is very expensive and suffers from portability issues. Investigators have been exploring marker-less imaging techniques e.g., Kinect integrated computer-based graphical user interfaces in stroke-rehabilitation such as tracking one's limb movement during rehabilitation. In our present study, we have developed a Kinect-assisted computer-based system that offered Human Computer Interaction (HCI) tasks of varying challenge levels. Execution of the tasks required one to use reaching and coordination skills of the upper limbs. Also, the system was Performance-sensitive i.e., adaptive to the individualized residual movement ability of one's upper limb quantified in terms of task performance score. We tested for the usability of our system by exposing 15 healthy participants to our system. Subsequently, seven post-stroke patients interacted with our system over a few sessions spread over 2 weeks. Also, we studied patient's mean tonic activity corresponding to the HCI tasks as a possible indicator of one's post-stroke functional recovery suggesting its potential of our system to serve as a rehabilitation platform. Our results indicate the potential of such systems toward the improvement of task performance capability of post-stroke patients with possibilities of upper limb movement rehabilitation.

## Introduction

One's ability to make adequate use of upper limbs is crucial for independently performing Activities of Daily Living (ADL) (Magermans et al., 2005). For executing ADL, one often needs to reach out and pick up an object while making coordinated hand movements. However, after stroke, such abilities become adversely affected and patients suffer from loss of dexterity, uncoordinated movement in upper limbs, etc., (Aqueveque et al., 2017). Consequently, stroke survivors become dependent on caregivers thereby adversely affecting their community life and ability to earn their own living. The situation becomes critical given the high prevalence of post-stroke cases. Globally, ~15 million people are affected with stroke every year (Green and King, 2010). Reports indicate that ~80% of stroke patients have upper limb dysfunction with ~30% of them regaining some functional recovery with rehabilitation in the first 6 months post-stroke (Turolla et al., 2013).

After stroke, the patients are commonly referred to physiotherapy units for rehabilitation. The conventional rehabilitation techniques require patients to have one-on-one sessions with clinicians. For developing countries like India, timely and adequate access to rehabilitation facilities including trained clinicians is restricted due to the limited availability of healthcare units and skilled clinicians. Given the high patient to doctor ratio particularly in developing countries like India (Deo, 2013), the post-stroke patients undergoing rehabilitation are often relieved from hospitals early. Also, in conventional rehabilitation, patients undergo exercises under one-on-one therapist supervision and the therapist's continuous presence often becomes a challenge with high patient load. Patients undergo repetitive exercises with limited variations that often become monotonous to them (Clarke, 2014). Thus, technology-assisted platforms built using the fundamentals learnt from conventional rehabilitation can offer intensive, interesting, and quantified rehabilitation while manipulating the tasks and thereby offering variations in challenge level. Also, such systems can alleviate the need for the continuous presence of a therapist by offering a powerful complementary tool in the hands of clinicians. For this, investigators have been exploring technology-assisted tools such as robot-based (Krebs et al., 2004; Hidler et al., 2005) and computer-based (Bao et al., 2013) platforms for rehabilitation.

Often, the robot-assisted platforms use goal-directed tasks such as following a pattern (Masia et al., 2009) that requires one to make a coordinated movement of upper limb. Again, researchers such as Wang et al. (2013) and Squeri et al. (2014) have used point-to-point reaching task to be performed under the assistance of a robot. The degree of task difficulty was varied by manipulating the range of movement needed to execute the task (Squeri et al., 2014). Added to offering tasks of varying challenges, researchers have used autonomous systems that can adapt to user's ability. One such robot-assisted autonomous system was used by Masia et al. (2009) and this system was capable of adaptively offering tasks of increasing difficulty once the user could satisfactorily complete a task of lower difficulty. The robot-assisted techniques, though powerful, often suffer from high cost, safety issues, no consideration of gravity while designing tasks, etc. (Hidler et al., 2005). Computer-based platforms integrated with optical motion capture systems (Hingtgen et al., 2006; Perez-Marcos et al., 2017) can offer another alternative. These studies have used marker-based techniques to track one's hand movement during a reaching task. Such systems used in stroke rehabilitation offer real-time processing and high accuracy of data. However, these systems often require a dedicated lab space, high set-up time and multiple cameras (Hingtgen et al., 2006). Often this is expensive and suffers from portability issues (Yang et al., 2014). In an attempt to develop cost-effective platforms, researchers have used single camera-based system (Sucar et al., 2010) that also uses markers. For example, the marker can be a ball held by the user (Sucar et al., 2010). Investigators have been exploring marker-less imaging techniques like Kinect integrated computer-based graphical user interface to track one's limb movement for functional assessment and rehabilitation (Bao et al., 2013; Roy et al., 2013; Sin and Lee, 2013; Webster and Ozkan, 2014; Kim et al., 2016; Gauthier et al., 2017; George et al., 2017; Liao et al., 2018). Researchers such as Kim et al. (2016) have used Kinect-assisted skeletal tracking, displayed on a computer-based graphical user interface for estimation of Fugl Meyer Assessment (FMA) score using the skeletal tracking data. Similarly, Kinect-assisted computer-based tasks have also been designed for upper-limb rehabilitation exercises. For example, Bao et al. have reported the use of Kinect-assisted computer-based upper limb rehabilitation therapy that aimed to improve the upper limb functionalities in post-stroke patients (Bao et al., 2013). Again, Liao et al. have used the Kinect-based system for postural tracking of a patient's upper limb for improving shoulder and elbow movements (Liao et al., 2018). Instead of offering only visual feedback, researchers have augmented such systems with auditory feedback (Sin and Lee, 2013). In one of the recent review articles (Webster and Ozkan, 2014), the use of Kinect coupled with computer-based graphical interface powered by Virtual Reality (VR) has been reported for upper limb rehabilitation exercises. As mentioned in this review paper, researchers such as (Chang et al., 2012; Webster and Ozkan, 2014) have proposed a Kinect-assisted system in which patients can manipulate virtual object through shoulder extension. Also, such systems have been used for guidance and correction of targeted upper extremity movements like shoulder abduction combined with elbow flexion (Webster and Ozkan, 2014) during exercise. Researchers have developed Kinect-assisted VR-based systems for upper limb rehabilitation in which post-stroke patients were asked to move their upper limb to steer a boat, pick up bottles, etc. in the VR world (Gauthier et al., 2017; George et al., 2017). In these studies, Kinect-assisted skeletal tracking was used to intermittently measure the functional ability of patients to monitor their functional progress during rehabilitative exercises. Further, researchers have presented Kinect-assisted VR-based game environments aimed toward improving the range of motion and other functionalities of patients (Borghese et al., 2012; Pastor et al., 2012). Though these studies have challenged the patient's functional ability while exposing them to the Kinect-assisted VR-based exercise platform, yet, in most of these cases, there was no task specific variation in the VR environment that can be motivating for patients. Additionally, none of these studies have investigated one's functional recovery along with physiological manifestation as gauged by clinicians using their expert eyes or physiological data acquisition. This is important, since physiological manifestations are often related to one's sensorimotor recovery (MacIntosh et al., 2008) that might be interesting to investigate while patients are exposed to a VR-based environment augmented with a Kinect device.

Investigators have been exploring the use of VR-based platforms augmented with Kinect. This is because, Kinect can offer several advantages such as cost-effectiveness, portability, ease of configuration, etc., (Lange et al., 2011). Though it has multiple advantages, the accuracy and precision for sensitive human-based applications are still under critical examination (Huber et al., 2015). As evident from literature, the error and imperfection in Kinect data can be due to the sensor, measurement setup, etc., (Khoshelham and Elberink, 2012). The sensor-related errors can be addressed through proper calibration, and the measurement setup-related error can be addressed by maintaining adequate lighting condition and controlling the distance of object from the sensor (Khoshelham and Elberink, 2012). However, given the advantages of using Kinect, investigators have been exploring its use in rehabilitation by augmenting it with computer-based systems. Computer-based platforms offer flexibility in design while making it easy to develop exercise tasks with variations for motivating the users (Bao et al., 2013). Thus, with Kinect-assisted computer-based systems, developing cost-effective, portable, and motivating exercise platforms with easy setup facilities has now become a reality. Also, literature review indicates the potential of such systems to address the issues of a reduced range of motion and coordination skill of post-stroke patients (Roy et al., 2013) thereby making it suitable for upper limb rehabilitation. Additionally, it would be interesting to explore the potential of such systems for adapting the task challenge level based on one's residual movement ability in an individualized manner.

Given the many advantages of using Kinect-assisted systems for monitoring one's limb movement during interaction with a computer-based graphical user interface, further exploration into its usage in adaptive exercise paradigms is warranted. Specifically, such a system can keep track of one's performance in an exercise task. Subsequently, the exercise platform can be made adaptive to one's residual movement ability (through task performance) while varying challenge level in an individualized manner. In our present study, we have developed a Kinect-assisted computer-based system powered with VR-based graphical user interface that offered Human Computer Interaction (HCI) tasks of varying challenge levels. Execution of the tasks required one to use reaching and coordination skills. Also, the system was Performance-sensitive being adaptive to the residual movement ability of one's upper limb quantified in terms of task performance score in an individualized manner. We tested for the usability of our system by healthy participants. Subsequently, post-stroke patients interacted with our system over a few sessions spread over 2 weeks and our results indicate the potential of such a system to contribute to upper limb rehabilitation.

The objectives of our study were 3-fold namely, to (i) understand the usability of our Kinect-assisted Computer-based HCI task platform among healthy individuals, (ii) know the views of post-stroke patients regarding the usage of our system, and (iii) understand the implications of interaction with the HCI task platform on the ability of post-stroke participants to perform hand movement over multiple exposures. Additionally, we wanted to understand the implications of these exposures on the physiology of post-stroke patients and the potential of our system to be used as a rehabilitation platform in future. This manuscript is organized as follows: section Materials and Methods discusses design of system; section Experimental Setup and Procedure describes experimental setup and procedure, section Result summarizes results, section Discussion presents the discussion, and section Conclusion presents conclusion.

## Materials and Methods

**Figure 1:**
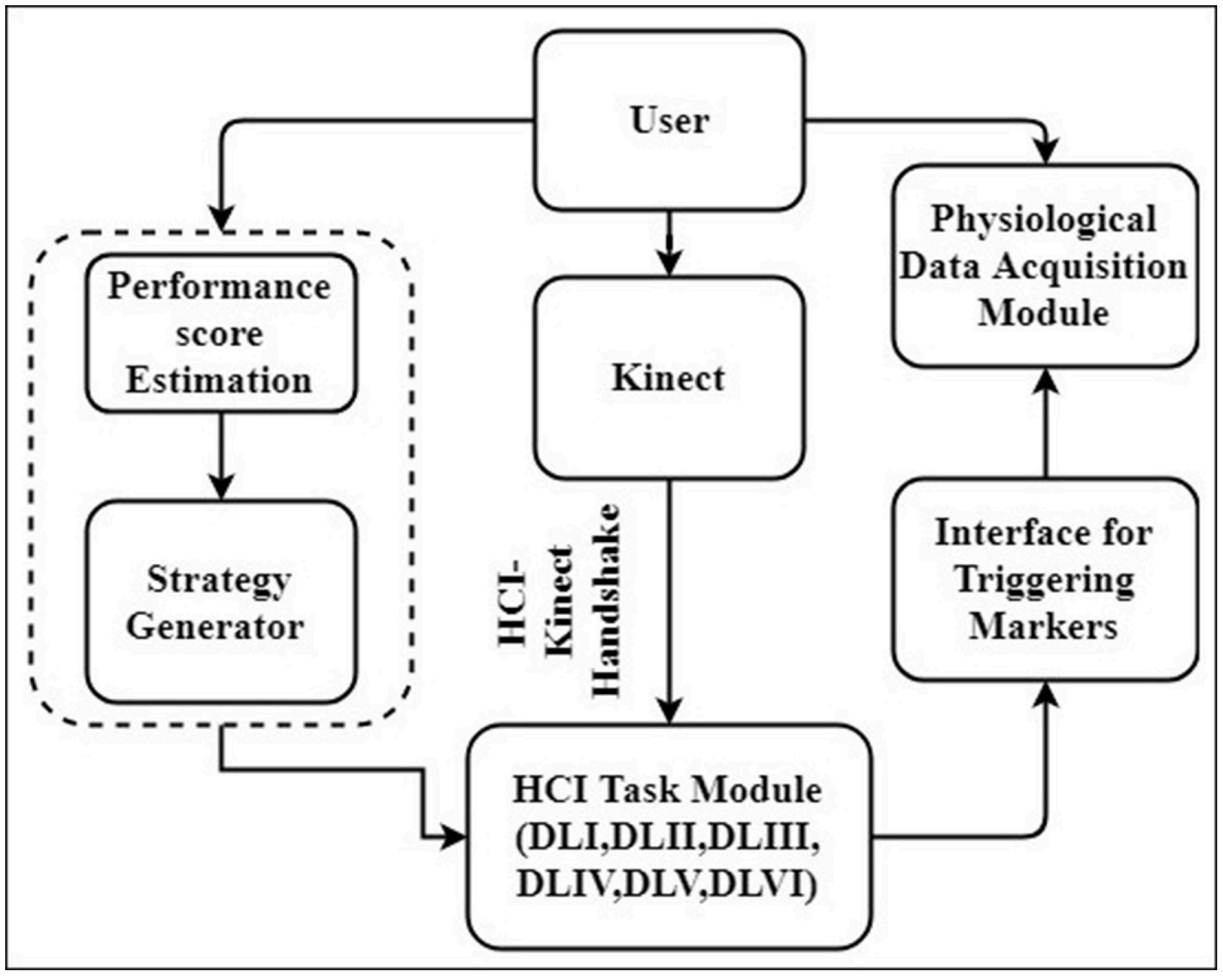
Block schematic of designed system.

Our system was composed of (1) Human Computer Interaction (HCI) Task, (2) HCI-Kinect Handshake (3) Strategy Generator, and (4) Physiological Data Acquisition modules. Figure 1 shows block schematic presentation of our system. As can be seen from this Figure, while the user (participant) performed upper limb exercise tasks, his hand position was picked up by a Kinect sensor interfaced with an HCI-based graphical user interface (using HCI-Kinect Handshake) presenting tasks of varying difficulty levels. Simultaneously, user's task performance score was computed by our system that in turn was used by a rule-based Strategy Generator to trigger switching of HCI-based tasks of varying difficulties. Additionally, the user's physiology was acquired by a Physiological Data Acquisition module, synched with the HCI-based platform by event markers. The data collection was carried out in two stages, namely Stage 1 and Stage 2. Stage 1 was used to ensure that our system was working as desired and understand the usability of our system. Stage 2 was used to understand the potential of our system to contribute to upper limb rehabilitation. Each Stage had two steps, namely Step 1 and Step 2. Step 1 was a calibration step in which we mapped one's hand movement from physical space to the virtual space. Step 2 involved interaction with the HCI task in which one's upper limb movement capability was challenged through tasks of varying difficulty levels. The Stages 1 and 2 were carried out with healthy and post-stroke participants, respectively.

### Human Computer Interaction (HCI) Task Module

We designed HCI tasks using the python based Vizard software (from Worldviz Llc.) and OpenCV library. The HCI tasks needed the participants to make coordinated movement in physical space (following specific trajectories displayed on the HCI monitor) coupled with stretching of upper-limb (i.e., reaching with flexed arm). Variation in task difficulty was also a part of the system design. The difficulty level depended on the (i) shape of the trajectories and (ii) extent of arm flexion required to complete a task.

#### Design of HCI Tasks

**Figure 2:**
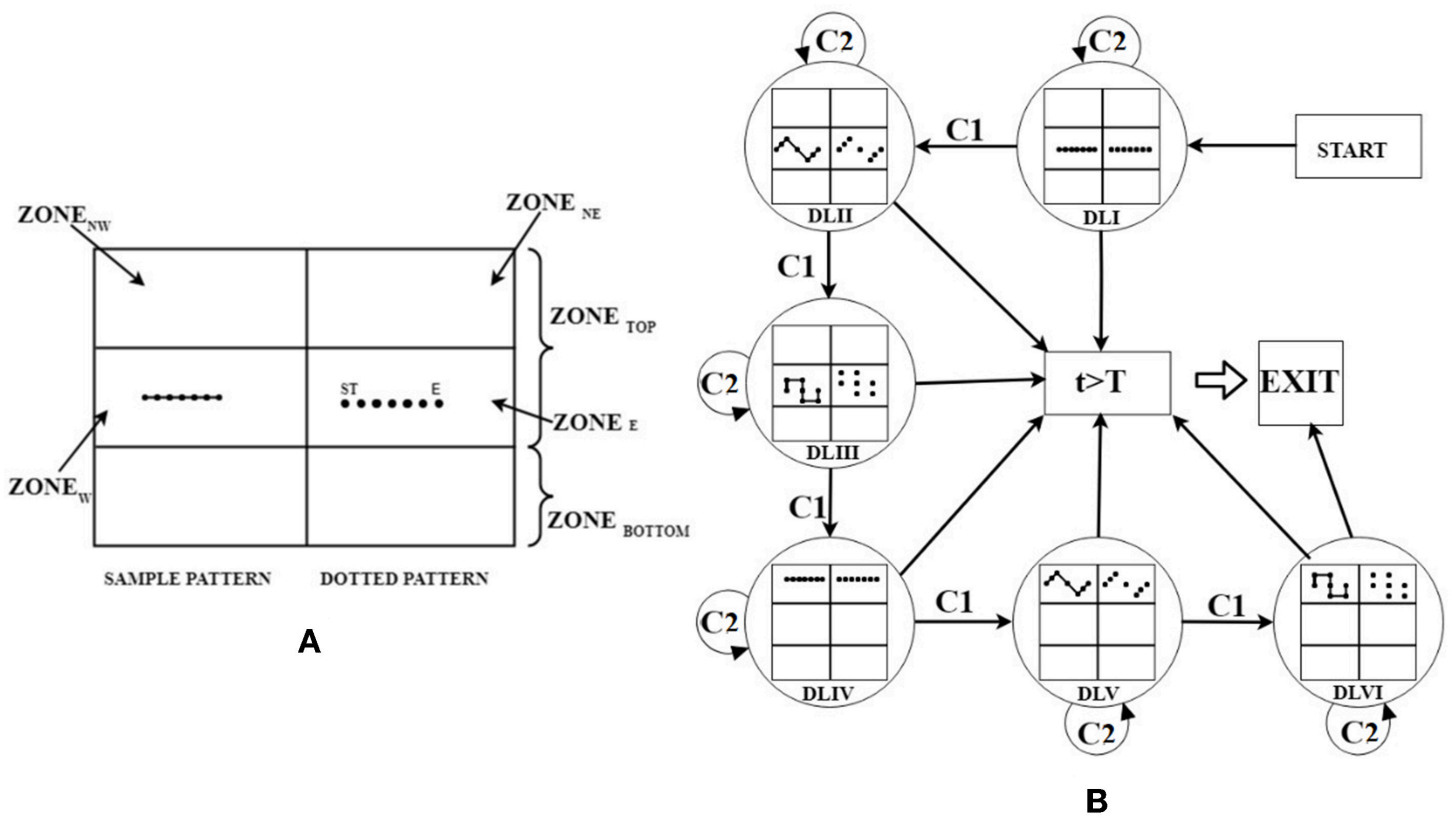
**(A)** Expanded view of GUI with DLI for Participant playing in Right hand, **(B)** state machine representation of strategy generator. ST, Start; E, End; t, Task execution duration; T, Maximum allowed duration; C1, Adequate Performance; C2, Inadequate Performance.

We designed a Graphical User Interface (GUI) for the HCI task. For each task, the GUI comprised of two sections, namely (i) Sample Pattern (solid lines) and (ii) Dotted Pattern (dots) displayed on both sides (Left and Right) of the Task monitor (Figure 2A). The Sample Pattern was used to serve as a Reference Pattern for the participant. The Dotted Pattern consisted of white colored dots (shown as black dots in Figure 2A) each of size 40 pixels in diameter displayed on a black colored background (shown as white background in Figure 2A for representation). The task required the participants to connect the dots of the Dotted Pattern (Getting idea from reference pattern). Henceforth, we refer the task of connecting the dots of the Dotted Pattern as a Sketching Task. Here, we have used three different patterns (e.g., Straight Line, Triangular, and Square wave patterns) those were to be followed by the participants while sketching the trajectories. Each pattern consisted of seven dots [one dot at start (ST) and end (E) of the trajectory with intermediate five dots]. For a Straight Line Pattern, the dots (spaced by 107 pixels and angular separation θ^0^ = 0^0^) were displayed (Figure 2A). The Triangular and Square wave Patterns were formed by displaying the dots at the vertices of the pattern [spaced by 107 pixels (θ^0^ = 60^0^) and 107 pixels (θ^0^ = 90^0^), respectively]. Please note that the number of dots and the spacing between dots were chosen as a typical case (can be changed based on requirement).

We chose these patterns taking inputs from rehabilitation studies (Wang et al., 2013) and the physiotherapist in our team to challenge participants' coordination capabilities. The Straight Line pattern was the easiest in terms of difficulty since it did not require one to change his/her hand orientation between the start and the end dots. The Triangular and Square patterns were of higher difficulty since these required one to change hand orientation between three to five times, respectively. Added to the coordination skills, one often needs reaching capability while performing activities of daily living. In fact, studies indicate that forward flexion exercise of one's arm (stretching of upper arm in plane parallel to frontal plane of body) often comes as an integral part of therapeutic regimen (Carr et al., 1985). To incorporate this, we designed HCI tasks that would need one's arm to be flexed by varying amounts in a plane parallel to the frontal plane. Thus, we segmented the HCI monitor into two parts namely Zone_TOP_, Zone_BOTTOM_ (Zone_TOP_: Zone_BOTTOM_ = 2:1 ratio) (Figure 2A). Here, we have considered only the Zone_TOP_ for the GUI. Subsequently, this Zone_TOP_ was subdivided into four sub-zones namely, Zone_NE_ (Top-Right), Zone_E_ (Middle-Right), Zone_NW_ (Top-Left), and Zone_W_ (Middle-Left). The Sample Pattern and Dotted Pattern were displayed in (i) Zone_E_, (ii) Zone_W_, (iii) Zone_NE_, and (iv) Zone_NW_. The choice of the zone for display of the Dotted Pattern was based on the left/right hand used by the participant to interact with the HCI task. For example, if a participant was asked to use the right hand for sketching the trajectory, then the Dotted Patterns were displayed in Zone_E_ and Zone_NE_ and the Sample Patterns were displayed in Zone_W_ and Zone_NW_. Vice-versa was the case for a participant using the left hand for HCI task. Sketching patterns in Zone_E_ and Zone_W_ needed one to flex his/her arm by 90^0^ ± 20^0^ and for Zone_NE_/Zone_NW_, the requirement was 120^0^ ± 20^0^ in a plane parallel to the frontal plane.

The task difficulty of the HCI tasks depended on the extent of arm flexion (specified in terms of Zone_E_/Zone_W_ and Zone_NE_/Zone_NW_) and the shape of the trajectories (i.e., Straight line, Triangular, and Square wave Patterns). The HCI tasks belonged to six difficulty levels (DLI–DLVI). Sketching of the Straight line, Triangular and Square wave Patterns in Zone_E_/Zone_W_ was considered to offer DLI, DLII, and DLIII tasks, respectively. Again, sketching of the Straight line, Triangular, and Square wave Patterns for Zone_NE_/Zone_NW_ were considered to offer DLIV, DLV, and DLVI tasks. One's hand position in the physical space was tracked by a Kinect sensor facing the individual and communicated to the Task computer by using HCI-Kinect Handshake Module (section HCI-Kinect Handshake Module). The sensed hand position corresponded to an avatar in the HCI environment. When the avatar collided with the dots of the Dotted Pattern displayed on the HCI monitor, the color of each dot changed from white to gray (with an auditory feedback). Corresponding to one's hand movement in the physical space, our system connected the consecutive dots with gray colored lines thereby sketching a pattern in each task. After a participant finished one task, the HCI task monitor displayed the performance score (section Computation of Performance Score) in percentage at center-top position of the monitor. Subsequently, based on the participant's performance score in a task, the Strategy Generator decided the difficulty level of the next task.

### HCI-Kinect Handshake Module

To execute the HCI tasks, the participant was asked to flex his arm in a plane parallel to the frontal plane. The position of the flexed arm in the physical space was captured using a Kinect xBOX 360 (from amazon.in/Microsoft-Kinect-Sensor-Xbox-360) that can perform skeletal tracking. This tracking device consists of one RGB camera, two depth sensing cameras, and one IR laser source. It generates images with 640 × 480 resolution (Roy et al., 2013). We used the Kinect mouse cursor application (Microsoft. archive.codeplex.com)^1^ that comes with the Kinect Windows SDK for designing the HCI-Kinect handshake module. We used this application for tracking the position of one's hand in physical space and representing that in the virtual space. For visualization, we integrated the participant's hand position with a paint brush (an avatar) in the HCI environment.

### Strategy Generator

The Kinect-assisted HCI system was programmed to autonomously offer tasks of six difficulty levels (DLI-DLVI) with the task difficulty being decided by a rule-based Strategy Generator. The Strategy generator offered tasks based on one's individualized task performance (section Computation of Performance Score) labeled as “Adequate” or “Inadequate”. The tasks of varying difficulty were offered using a state machine representation (Figure 2B). One's performance was labeled as “Adequate” (Condition C1) if the performance score was ≥70%, else, it was labeled as “Inadequate” (Condition C2). Here, the threshold for the performance score was taken as 70% as an initial approximation (can be changed) since literature indicates 70% as the average initial exercise performance for outpatient clinics (Jack et al., 2010). This threshold has been used in technology-assisted studies (Metzger et al., 2014; Young et al., 2014). In the study by Young et al. (2014), researchers have used the threshold of 70% for tuning the difficulty level of tasks offered by computer-assisted skill learning platform for post-stroke patients with upper extremity movement disorder. Unlike our present study in which the task difficulty was modulated based on one's performance, Young et al. displayed the task on a Brain Computer Interface and their system modulated the tasks based on patient's physiological data. In the study by Metzger et al. (2014), 70% criteria was used while offering upper limb rehabilitation on grasping skills to post-stroke patients using a robot with two degrees of freedom. Unlike our study in which post-stroke patients were asked to carry out a computer-based task that needed them to lift their hands against gravity, here (Metzger et al., 2014) the robot was planar type without any consideration of the effect of gravity. If a participant's performance score in an HCI task was “Adequate,” then the Strategy Generator selected “increase difficulty” mode that offered a task of higher difficulty level. In contrast, if the performance score was “Inadequate,” then the Strategy Generator selected “same difficulty” mode that offered a task of same difficulty level as the previous task. While our system kept a record of the task performance, for “Inadequate” performance, instead of reducing the task difficulty level, our system chose the “same difficulty” mode with an aim to impart skill training of the participant. Upon practice, once the participant acquired improved skill (performance score ≥70%, i.e., “Adequate”), then the participant was challenged by a task of increased difficulty. The HCI tasks continued till the end of a pre-defined task duration or once a participant had achieved “Adequate” performance in a task belonging to DLVI.

#### Computation of Performance Score

Our system was designed to be performance-sensitive. While our participants interacted with tasks of varying difficulty levels, we computed two measures of task performance namely, *Perf*_*SCORE*_ (for each difficulty level) and weighted normalized performance score (*Perf*_*NORM*_). The *Perf*_*NORM*_ was computed (for comparative assessment) to account for a varying number of tasks belonging to each difficulty level being executed by different participants.

One's task performance score depended on (i) Metric1: the number of dots (Dotted Pattern) touched by the avatar (by participant's hand movement) (*Dot*_*NO*_) on the GUI (HCI screen) and (ii) Metric 2: the length of the trajectory sketched by the participant's hand (*Traj*_*LENGTH*_). As far as Metric 1 was concerned, we computed the ratio (*Dot*_*R*_) of the *DOT*_*NO*_ to the total number of dots in a Pattern (*Dot*_*TOTAL*_) using Equation (1). As far as Metric 2 was concerned, we found that the total length of the trajectory sketched by even healthy participants i.e., Optimal Trajectory Length (*Traj*_*LENGTH*_*OPT*_) (measured using a pilot study incorporating age-matched groups) was mostly greater than the actual length of the Pattern formed by joining the dots with straight line segments. Subsequently, we computed the ratio (*Traj*_*Ri*_) of the *Traj*_*LENGTHi*_ to *Traj*_*LENGTH*_*OPTi*_ for task of each difficulty level *(i)* using Equation (2).

(1) $$\begin{array}{lll} DOT_{R} & = & \frac{DOT_{NO}}{DOT_{TOTAL}} \end{array}$$

(2) $$\begin{array}{lll} Traj_{Ri} & = & \frac{Traj_{LENGTHi}}{Traj_{LENGTH\text{\_}OPTi}};i=1,2,3,4,5,6 \end{array}$$

Again, based on the pilot study, we found the Minimum (*Traj*_*Ri*_*MIN*_) and Maximum (*Traj*_*Ri*_*MAX*_) values of *Traj*_*Ri*_ corresponding to the task of each difficulty level *(i)*. If a participant's *Traj*_*LENGTHi*_ corresponding to a *Traj*_*Ri*_ value was within the limits (*Traj*_*Ri*_*_*_*MIN*_ and *Traj*_*Ri*_*_*_*MAX*_), the contribution of Metric 2 to the estimation of performance score was not penalized (Case 1). Else, depending on the extent of overshoot/undershoot of *Traj*_*LENGTHi*_ beyond the *Traj*_*LENGTHi*_*_*_*OPTi*_, a penalty was imposed (Cases 2 and 3) as seen from Equation (3). The extent of allowable (with limited penalty) overshoot/undershoot was chosen to be 20% as a typical case and this can be varied based on task design. Finally, our HCI system evaluated the overall Performance Score (*Perf*_*SCORE*_) while considering both the Metric 1 and Metric 2 i.e., *Dot*_*R*_ and *Traj*_*Ri*_ with equal weightage (= 0.5) for each task (Equation 4).

(3) $$\begin{array}{l} Case1:\;if\;Traj_{Ri_{MIN}}<\;Traj_{Ri}<Traj_{Ri_{MAX}},\\ \text{then the case }-\text{ specific}\;\;Traj_{Ri}=1. \end{array}$$

(4) $$\begin{array}{l} Case2:if\;0.8\;Traj_{Ri_{MIN}}<Traj_{Ri}<1.2Traj_{Ri_{MAX}},\\ \text{then the case }-\text{ specific}\;Traj_{Ri}=0.5.\\ Case3:\;\;\text{case }-\text{ specific}\;Traj_{Ri}=0\;(otherwise).\\ Perf_{SCOREi}=100*((0.5*Dot_{R})+(0.5*Traj_{Ri}));\;i=1,2,3,4,5,6 \end{array}$$

Additionally, we computed the *Perf*_*NORM*_ while considering the task performance in different tasks belonging to varying difficulty levels.

(5) $$\begin{array}{l} Perf_{NORM}=\frac{\sum_{i=1}^{6}w_{i}\times Perf_{SCORE_{i}\;}}{\sum_{i=1}^{6}w_{i}}\;,w_{1}=1,w_{2}=2,\ldots \;w_{6}=6. \end{array}$$

Here, *w*_*i*_ is the weight assigned to a task belonging to a particular difficulty level and *i* represents the difficulty level.

### Physiological Data Acquisition Module

We acquired Electrodermal Activity (EDA) signal of post-stroke participants during Stage 2 of the study using Biopac MP150 (Biopac Systems Inc.). The EDA signal was acquired in the wireless mode in a Data Logger Computer interfaced with the HCI Task computer during baseline and HCI tasks. The signals were acquired at a sampling rate of 1,000 Hz. The EDA signal representing one's skin conductivity, was acquired by using sticky use-and-throw electrodes with Ag/AgCl gel from the distal phalange of the index and ring fingers of one's hand. We chose to study the EDA signal, since EDA can be used as an objective measure of effort put in by an individual during the execution of a task (MacIntosh et al., 2008; Clarkson et al., 2010). The EDA signal was processed to extract the Tonic Mean (*Tonic*_*MEAN*_). Literature review indicates that the tonic component can be related to a task being executed and the perceived challenge by the post-stroke patient (Chatterjee et al., 2018) with a reduction in tonic activity indicative of functional recovery (Clarkson et al., 2010).

In order to nullify the effect of baseline recording, we extracted the tonic mean both during baseline (*Tonic*_*MEAN*_*Baseline*_) and each task (*Tonic*_*MEAN*_) in a time-synched manner. For triggering markers to specify start and end of the HCI tasks, we used an Arduino DUE-based setup (www.arduino.cc/en/Guide/ArduinoDue) that interfaced the HCI Task computer to the Data Logger Computer through a Tx/Rx pair of the Arduino DUE. Since the post-stroke participants were offered multiple exposures to our HCI task platform on different days during Stage 2 of the study, we computed the change in Tonic Mean with respect to the Baseline (Δ*Tonic*_*MEAN*_) in order to nullify the effect of day variability using Equation (6).

(6) $$\begin{array}{l} \Delta Tonic_{MEAN}=\Delta Tonic_{MEAN}-\Delta Tonic_{MEAN\_Baseline} \end{array}$$

## Experimental Setup and Procedure

### Participants

Fifteen healthy participants [H1–H15; mean (SD) = 43 (14) years] volunteered from our institute and the neighborhood during Stage 1 of our study. We first conducted our study with these healthy participants to ensure that our system was working as desired and to understand the usability of our system. This was followed by Stage 2 in which seven post-stroke [S1–S7; mean (SD) = 53 (14) years] participants took part. The idea was to understand the potential of our system to contribute to upper limb rehabilitation. The stroke participants, undergoing treatment in a local hospital enrolled through physiotherapist's referral. For each post-stroke participant, Fugl-Meyer^2^ Assessment of Sensorimotor Recovery after Stroke (FMA) (https://neurophys.gu.se) was carried out by a clinician. The inclusion criteria were (1) post-stroke period >6 months (2) ability to follow instructions (3) ability to see the HCI monitor from a distance of ~1.5 m, and (4) FMA score for Upper Extremity >31 (since our tasks required participants to do elevated hand posture by lifting the hand against gravity) that was measured by a physiotherapist in our team. Patients with recent surgery (<5 months) and having pace makers were excluded from the study. Our study followed institute ethics. The participant characteristics are shown in Table 1. As far as the Post-stroke patients were concerned, from the FMA scores, we can say that S1, S3, S4, S6, and S7 can be considered to have “limited capacity” in the upper extremity (with S1 and S7 being in the upper side of the spectrum toward the “notable capacity” range) and participant S2 had “notable capacity” in the upper extremity (Singer and Garcia-Vega, 2017).

**Table 1:** Participants' characteristics.

**ID**	**Age(y)**	**Paretic side**	**PSP(y)**	**Number of session**	**FMS**
P1	26–30	NA	NA	1	NA
P2	50–55	NA	NA	1	NA
P3	50–55	NA	NA	1	NA
P4	66–70	NA	NA	1	NA
P5	36–40	NA	NA	1	NA
P6	50–55	NA	NA	1	NA
P7	30–35	NA	NA	1	NA
P8	26–30	NA	NA	1	NA
P9	20–25	NA	NA	1	NA
P10	26–30	NA	NA	1	NA
P11	60–65	NA	NA	1	NA
P12	60–65	NA	NA	1	NA
P13	40–45	NA	NA	1	NA
P14	46–50	NA	NA	1	NA
P15	26–30	NA	NA	1	NA
S1	56–60	R	0.7	3	45
S2	56–60	L	6	3	49
S3	26–30	L	3	3	35
S4	70–75	R	5	2	32
S5	50–55	R	–	3	–
S6	46–50	L	4	3	39
S7	56–60	R	11	2	43

*M, Male; F, Female; PSP, Post Stroke Period; FMS, Fugl Mayer Score; L, Left side; R, Right Side; P1-P15, Healthy Participant; S1–S7, Stroke Participant; NA, Not Available; y, years*.

### Experimental Setup

**Figure 3:**
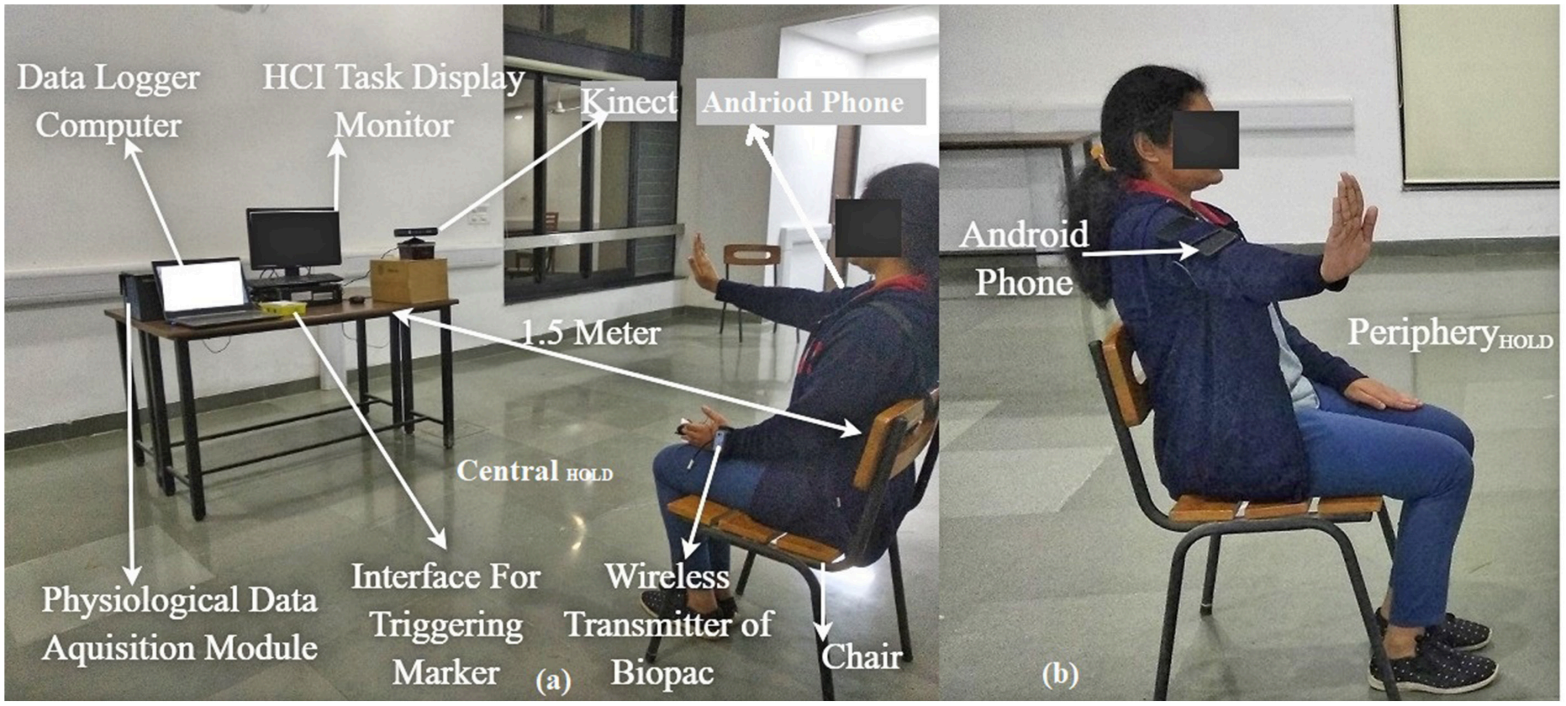
Experimental Setup **(a)** Participant having hand posture Central_HOLD_, **(b)** participant having hand posture Periphery_HOLD_ during calibration. Written and informed consent is taken from the participant toward publication of the image.

This study was conducted in two stages (Stages 1 and 2) with each stage having two steps namely Step 1 and Step 2. Step 1 being a calibration step, consisted of an HCI task computer [PC1; Intel(R) core(TM) i7-4770 CPU with 23” monitor having resolution of 1600 × 900 pixels), android phone (Redme Note 5 Pro), Velcro belts, a chair and Kinect (Figure 3a). As can be seen from this Figure 3a, the android phone (with orientation sensor application program adopted from “Physics Toolbox Sensor Suite” (Vieyra and Vieyra, 2018) was mounted on one's upper arm with the help of a Velcro belt and the azimuth angle recorded by the phone was used to find the range of horizontal abduction and adduction of arm corresponding to the Central_Hold_ and Periphery_Hold_ (Figure 3b) positions in an individualized manner. The range of horizontal abduction and adduction of the arm was mapped to the horizontal span of ~700 pixels of the HCI task monitor. The Step 2 used for interaction with the HCI task consisted of the HCI Task monitor, Kinect, a chair and a velcro belt in Stage 1 along with a physiological data acquisition module in Stage 2 of the study (Figure 3A). Additionally, in Stage 2, the experimental setup had a Data Logger Computer to record the physiological data acquired using the physiological data collection module. Since we were interested to study the implication of exposure to our system on the tonic mean of post-stroke patients, we took care of confounding factors such as variation in room temperature that can contribute to one's thermoregulatory sweating. For this, the room (data collection room) temperature was kept constant. The chair (for the participant) was positioned at a distance of 1.5 m from the Kinect and the Kinect itself was mounted on a table ~1.3 m above the ground, similar to other studies (Tao et al., 2013). The study room was uniformly lit.

### Procedure

The study required nearly 40 min per session from each volunteer. When the participant came in to the study room, he/she was offered to sit on a chair and asked to relax for about 5 min during each session. During this time, the experimenter explained what the participant was expected to do in the study and showed the experimental setup along with the GUI displaying the Sample and Dotted Patterns. The experimenter used a visual schedule to explain the HCI tasks to the participants. Also, the experimenter confirmed that the participant could see the GUI clearly from a distance of 1.5 m. The experimenter told participants that were free to quit from the study at any point if they felt strained and uncomfortable in interacting with the system. Again, since we were interested to study one's physiological manifestation in terms of electrodermal activation that can be susceptible to variations with physical strain (Boettger et al., 2010) during task execution, we tried to minimize such an effect by telling the patients to do the HCI tasks while sitting on a chair and taking rest between the tasks. The Stage 1 was meant for healthy participants and this was carried out at the research lab situated within our institute premises. For Stage 1 of our study, the experimenter determined the hand dominance of the participant using Edinburgh Handedness Inventory (Oldfield, 1971). Again, for Stage 1, we offered an exposure for one session. The Stage 2 was meant for post-stroke participants and this was carried out within a clinical setting inside the hospital where the patients were undergoing therapy. For Stage 2 of our study, the clinician in our team assessed the participant's FMA score and also checked the medical records to match the inclusion criteria. For Stage 2, each participant was offered exposure to our system over multiple sessions distributed over multiple days. Then the experimenter told the participant that he/she will be asked to use one arm at a time to interact with the HCI task platform. For Stage 2, the experimenter told the participants that after the first session, they would be asked to use only their paretic hand to interact with the HCI task platform. Once the participant understood the task, the experimenter administered the signing of the consent form. For Stage 2, the experimenter attached the light-weight EDA sensors on the index and ring fingers of the non-paretic hand of the participant. Upon getting a confirmation from the participant regarding his/her participation in the study, the experimenter asked the participant to sit straight on the chair and keep his back resting to the back-rest of the chair while facing the Kinect sensor. Also, the participant was told that he/she would be asked to continue to maintain the same sitting posture throughout the task. For Stage 1 of the study, Step 1 was carried out first and this was followed by Step 2 that lasted for ~20 min. During Step 1, the participant was told that he/she would see a “+” sign on the HCI Task monitor indicating that his hand has been captured by the Kinect sensor. During the Step 1, the participant was asked to stretch his/her arm (~90^0^ forward Flexion as measured by the android-based application; Figure 3a) when the “+” sign appeared at the Central_Hold_ position of the Task monitor. Then, he/she was asked to perform horizontal adduction of the arm as much as possible (staying inside the Field of View of the Kinect) while this was registered by the “+” sign displaced horizontally by a distance of *x* pixels from the Central_Hold_ position (either to the left or right of the Central_Hold_ position depending on the hand used). The distance *x* being a function of the hand length and the amount of adduction angle at the shoulder joint was mapped to ~700 pixels to achieve the Periphery_Hold_ position thereby making the calibration individualized. The adduction angle was recorded by the experimenter using the android phone-based orientation sensor application program. After Step 1, the experimenter asked the participant whether he/she was ready to start interacting with the HCI tasks (Step 2). During Stage 2 of the study, the experimenter followed Step 1 and Step 2 similar to that in Stage 1. However, since the participants in Stage 2 were post-stroke patients, mostly the experimenter helped the participants during Step 1 (of Stage 2). Again, before starting Step 2 (of Stage 2), the experimenter checked to see whether the physiological signals were acquired properly in the Data Logger Computer. Step 2 started with a baseline recording of the EDA signal for 3 min with the participant relaxing while sitting on the chair. Post baseline recording, the participant was asked to interact with the HCI task platform (Step 2). During Step 2 of Stage 2 of each session, each participant was asked to interact with the HCI tasks using three trials. While the first two trials were considered as practice trials, the data was recorded during the last trial for each task. While presenting our findings, we would use the data of the last trial of session 1 (Exp_1_ *henceforth*) and that for the last trial of the last session (Exp_L_ *henceforth*) for each post-stroke participant.

### Statistical Analysis

Given the limited sample size, we carried out normality test, namely Shapiro-Wilkes test of normality (Gaddis and Gaddis, 1990) and the data were found not to be normally distributed. Thus, we carried out a non-parametric statistical test such as the Friedman Test (Gaddis and Gaddis, 1990), Wilcoxon rank-sum test (Gaddis and Gaddis, 1990) and Wilcoxon Signed Rank test (Gaddis and Gaddis, 1990) (*p* ≤ 0.05). For statistical tests on the performance score of healthy participants across varying difficulty levels, we carried out a dependent sample Friedman Test for multiple groups of data (i.e., difficulty levels). While the healthy and post-stroke participants interacted with a varying number of tasks belonging to each difficulty level, we wanted to understand whether their functional abilities in terms of weighted normalized performance (*Perf*_*NORM*_) were statistically different. For this, we carried out independent sample Wilcoxon rank-sum test on the normalized performance of the healthy and post-stroke group while considering the data for Exp_1_. Also, we wanted to understand whether the improvement in the *Perf*_*NORM*_ score of the post-stroke participants interacting with our system from Exp_1_ to that in Exp_L_ was statistically significant. For this we carried out dependent sample Wilcoxon Signed Rank test on their *Perf*_*NORM*_ score. Similar was the case for the statistical analysis on the mean tonic activity of the post-stroke patients during Exp_1_ and Exp_L_.

### Post-study Survey Questionnaire

To understand the usability of a task platform, it is necessary to measure the user's experience. After the participants finished interacting with our Kinect-assisted HCI task platform, we wanted to measure their experience in terms of understandability, efficacy of feedback and interestingness (Liu et al., 2017). For this, we framed survey questionnaires taking idea from the User Suitability Evaluation Questionnaires proposed by Gil-Gómez et al. (2017). We framed six questions (Q1–Q6) and used a 5-point scale ranging from 1 (Strongly Disagree) to 5 (Strongly Agree). The questions were “Did you face any difficulty in understanding the tasks?” (Q1), “Did you find the task interesting?” (Q2), “Was the feedback such as Performance Score, Audio-visual display of the tasks useful to you?” (Q3), “Do you want to refer any of your acquaintances to this study?” (Q4), “Do you want to participate again in this study?” (Q5) and “Do you think that you can benefit by using such a system in future?” (Q6). The questions Q1–Q3 and Q1–Q6 were asked during Stage 1 and Stage 2 of the study, respectively.

## Result

In our study, the task was to sketch patterns on an HCI monitor while connecting dots of a Dotted Pattern displayed on the monitor. Our study comprised of two stages namely Stage 1 (healthy participants) and Stage 2 (post-stroke participants) with each stage consisting of Step 1 and Step 2. The Step 1 was for calibration (section Experimental Setup) and Step 2 was for task execution. Here we present our findings while following Step 2 of both the Stages 1 and 2 along with post-study survey to obtain participants' feedback in both the stages.

### Post-study Feedback of Stage 1 of the Study

**Figure 4:**
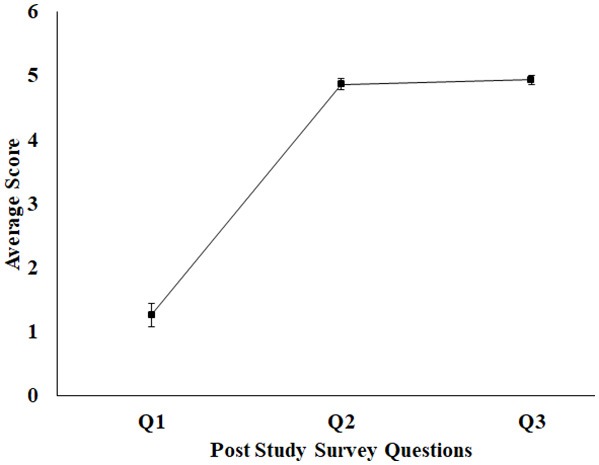
Average feedback score for healthy participant (1–5 scale). Error bars represent standard error. 1, Strongly Disagree; 2, Disagree; 3, Neutral; 4, Agree; 5, Strongly Agree.

After the healthy participants finished interacting with the Kinect-assisted HCI task platform, a post-study survey [Q1–Q3 (section Post-study Survey Questionnaire) and 5-point scale] was administered by the experimenter and the survey results in form of average score for each question (standard errors) are shown in Figure 4. It can be seen from Figure 4, the healthy group did not face any difficulty in understanding the tasks and most of them “Strongly Agreed” on the HCI tasks being interesting. When asked about the Reference Patterns, all of them said that it helped them understand the pattern that they need to sketch. As regards to the sketched pattern (Dotted-Pattern), all of them said that the audio-visual feedback by the task platform while the paintbrush touched a dot in the Dotted Pattern (section Design of HCI Tasks) corresponding to their hand movement offered an immersive feel to them. As far as the feedback on the Performance Score was concerned, most of them mentioned that the score helped them to understand how they were performing in each task and motivated them to try out the next task.

### Group Average Performance of Healthy Participants for Stage 1 of the Study

**Figure 5:**
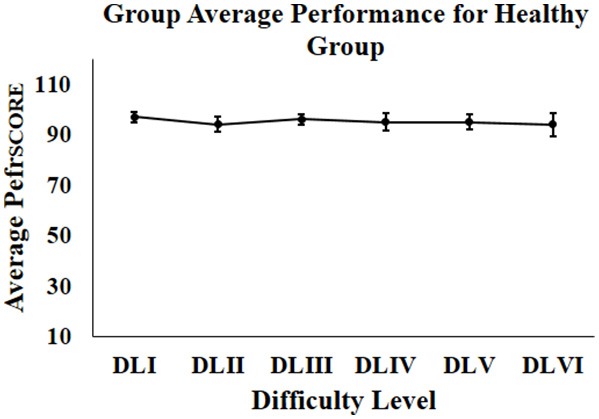
Group average performance of healthy participant. error bars represent standard error.

We wanted to test our Kinect-assisted HCI task platform with healthy participants. Our results indicate that all the healthy participants scored “Adequately” in the first task in each difficulty level. Figure 5 shows the % average performance (Standard Error) of healthy participants in all difficulty levels. From Figure 5, we can see a nearly ceiling effect in the performance score of the healthy participants irrespective of the task difficulty level, as expected. To test the data for statistical significance, we used dependent sample Friedman test on the performance scores of the healthy participants across varying difficulty levels (DLI to DLVI). The results were not statistically different, showing that there exists no significant difference between the performance of the healthy subjects over varying difficulty levels.

### Post-study Feedback of Stage 2 of the Study

**Figure 6:**
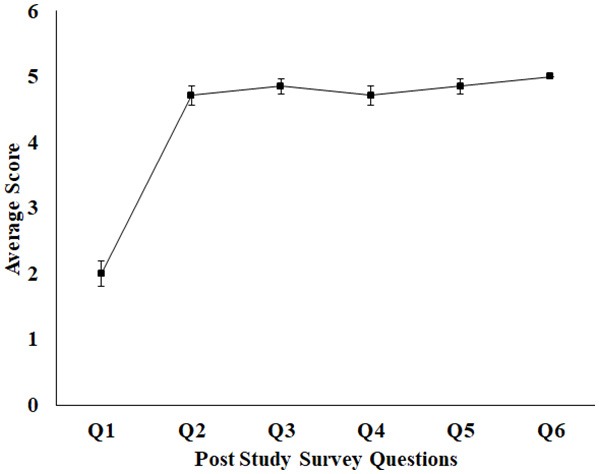
Average feedback score for post-stroke participant (1–5 scale). Error bars represent standard error. 1, Strongly Disagree; 2, Disagree; 3, Neutral; 4, Agree; 5, Strongly Agree.

Once the post-stroke participants finished interacting with the HCI task platform at the end of the last session, the experimenter administered a post-study survey [Q1–Q6 (section Post-study Survey Questionnaire)] shown in Figure 6. Figure 6 shows the average score for each question with standard errors bars. It can be seen from the Figure 6 that the post-stroke group did not face any difficulty in understanding the tasks, similar to the healthy group. Also, all of them reported “Strongly Agree” on the other five questions. With regard to the interestingness and the usefulness of the feedback, the response of the post-stroke group is same as the healthy group. All the post-stroke participants expressed their interest in participating again and asked about the future possibilities. Though the number of sessions were limited (based on patients' availability), yet, all the participants expressed that they think that they would benefit by using our system. When asked to elaborate the reason behind their thought, most of them said that even with limited exposure to our task platform, they are hopeful that exercising with our system can improve the coordination, and reaching skills in their upper limbs.

### Comparative Analysis of Average Performance of Post-stroke Participants in Tasks of Varying Difficulty Levels During Exp_1_ and Exp_L_ of Stage 2 of the Study

**Figure 7:**
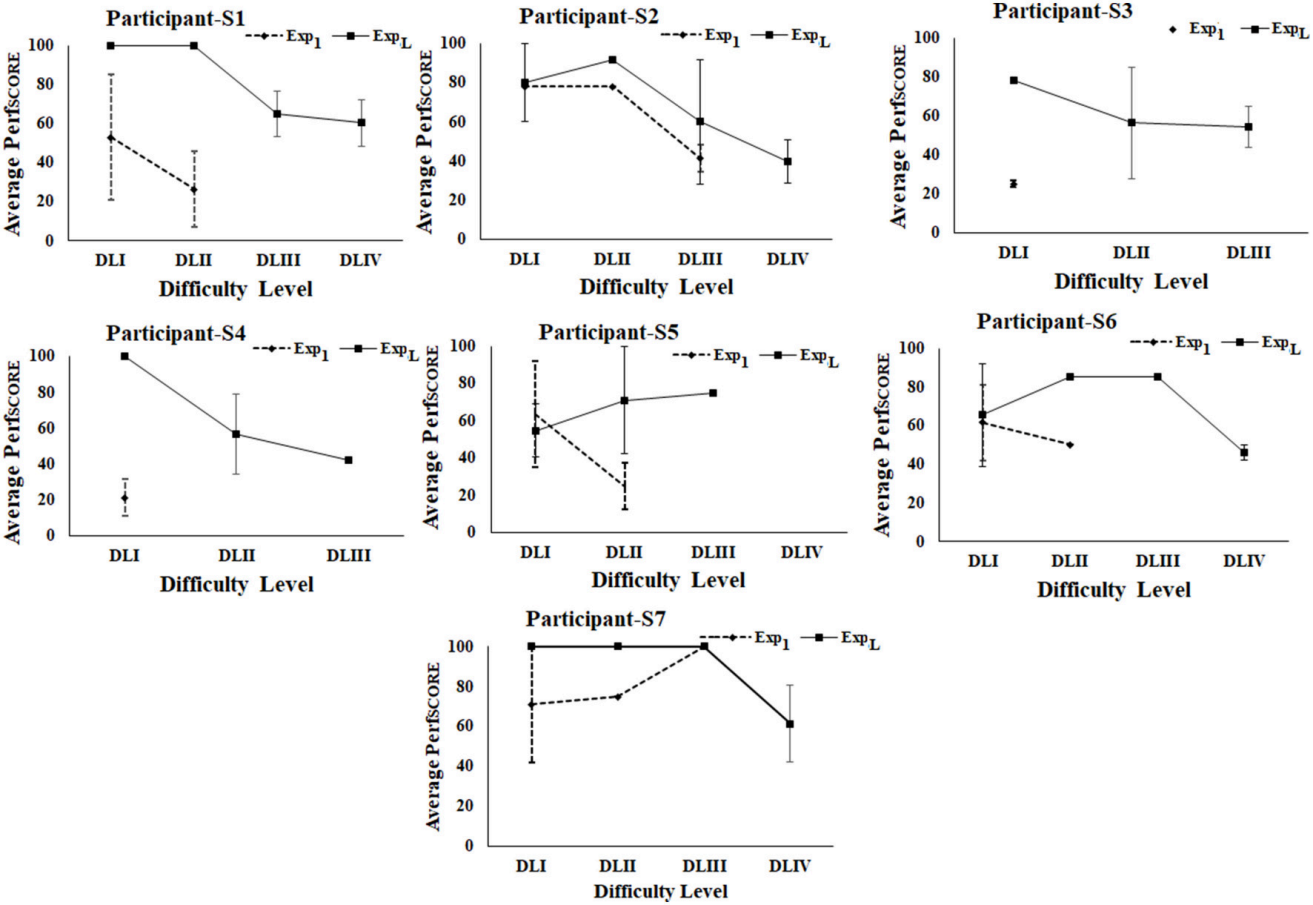
Comparative analysis of average performance of stroke participants. Error bars represent standard error. Exp_1_, First Exposure; Exp_L_, Last Exposure.

During Stage 2 of the study, we wanted to understand the implications of interaction with the Kinect-assisted HCI task platform on the upper limb-related coordination skill of post-stroke participants. The participants were offered the performance-sensitive HCI tasks of varying challenges based on their ability to make coordinated movement. Once a participant acquired “Adequate” performance score in a task belonging to a difficulty level, our system offered a task of higher difficulty. To understand the implication of multiple exposures (to our system) on one's performance (*Perf*_*SCORE*_; section Computation of Performance Score), we carried out a comparative analysis of *Perf*_*SCORE*_ in tasks (Figure 7) belonging to each difficulty level during Exp_1_ and Exp_L_. Figure 7 shows average *Perf*_*SCORE*_ and standard errors for each difficulty level. From Figure 7, we find that all the participants performed better during Exp_L_ as compared to Exp_1_ in terms of (i) improved % average *Perf*_*SCORE*_ in tasks of a difficulty level and (ii) ability to reach to tasks of higher difficulty level. Such an increase in performance for all participants (except S5) in tasks of DLI can be possibly attributed to improvement in coordination ability (in following the trajectory of the Dotted Pattern). In the case of S5, we see a small decrease (Δ = ~9%) in the % performance in the task of DLI during Exp_L_ compared to Exp_1_. We performed a deeper investigation which found that during Exp_1_, S5 could achieve “Adequate” performance in the second task while he could achieve “Adequate” performance in the sixth task during Exp_L_. The experimenter reported that though S5 needed to interact with five tasks for each DLI before getting an “Adequate” performance score during Exp_L_, yet his score even in the first of the five tasks (for DLI) was higher compared to his score in the first task belonging to DLI during Exp_1_. When the experimenter asked him for the reason behind such a change in his performance during Exp_L_, he told that he was feeling tired and could not hold his hand against gravity during the intermediate three tasks. Again, in contrast to Exp_1_, we find from Figure 7 that four of the seven post-stroke participants could reach DLIV during Exp_L_ and the rest could reach DLIII during Exp_L_. However, improvement in one's performance capability not only depends on his/her ability to interact with tasks of increased challenge, but also depends on how fast (i.e., within fewer tasks) one was able to achieve “Adequate” performance in a task of a particular challenge level before going to a task of higher difficulty level i.e., the trajectory of task progress.

### Comparative Analysis of Task Progression Pattern of Post-stroke Participants in Tasks of Varying Difficulty Levels During Exp_1_ and Exp_L_ of Stage 2 of the Study

**Figure 8:**
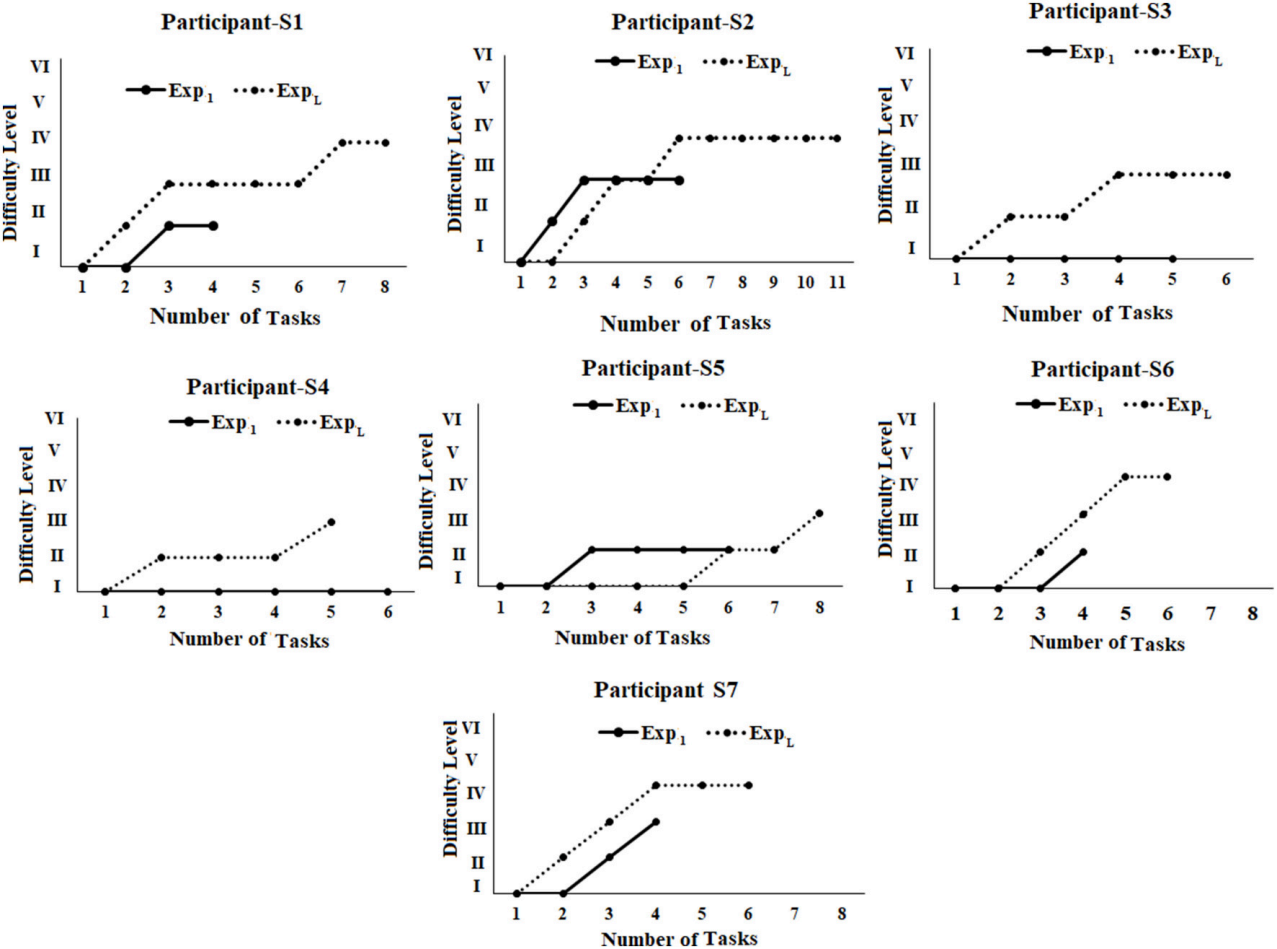
Comparative analysis of task progression pattern of post-stroke participants. Exp_1_, First Exposure; Exp_L_, Last Exposure.

We wanted to understand whether our HCI task platform had any implication on task progression pattern of the participant from one difficulty level to another. Figure 8 shows the task progression pattern of each post-stroke participant during Exp_1_ and Exp_L._ From Figure 8 we could see [in all the participants (except S2 and S5)] a comparatively smoother transition from lower to higher difficulty levels with the number of tasks needed before achieving “Adequate” performance in a particular difficulty level for Exp_L_ compared to Exp_1_. Participant S2 needed two tasks in DLI before moving to DLII during Exp_L_ compared to only one task in DLI during Exp_1_. However, a deeper analysis revealed that unlike Exp_1_, during Exp_L_, S2 was able to reach the DLIV task, and also achieved “Adequate” performance in the last task belonging to DLIV and had to quit the HCI task since the allotted duration was over. Again, for S5, we see that unlike Exp_1_, during Exp_L_, S5 needed five tasks to achieve “Adequate” performance in DLI. When he was asked the reason for such a performance at the end of his interaction with our task platform after Exp_L_, he expressed that he was tired and discontinued in between tasks (section Comparative Analysis of Average Performance of Post-stroke Participants in tasks of varying Difficulty Levels during Exp1 and ExpL of Stage 2 of the study). However, he could reach to DLIII during Exp_L_ unlike that during Exp_1_. Please note that S3, S4, and S6 could perform mostly in DLI during Exp_1_ that can be possibly attributed to their “limited capacity” of upper extremity as specified by the FMA scores (section Participants). However, multiple sessions with our performance-sensitive HCI task platform could elicit improved performance even in these participants as can be seen from Figure 8. Given a small sample size, we do not intend to generalize our observations.

### Comparative Analysis of Weighted Normalized Performance Score (*Perf_*NORM*_*)

**Figure 9:**
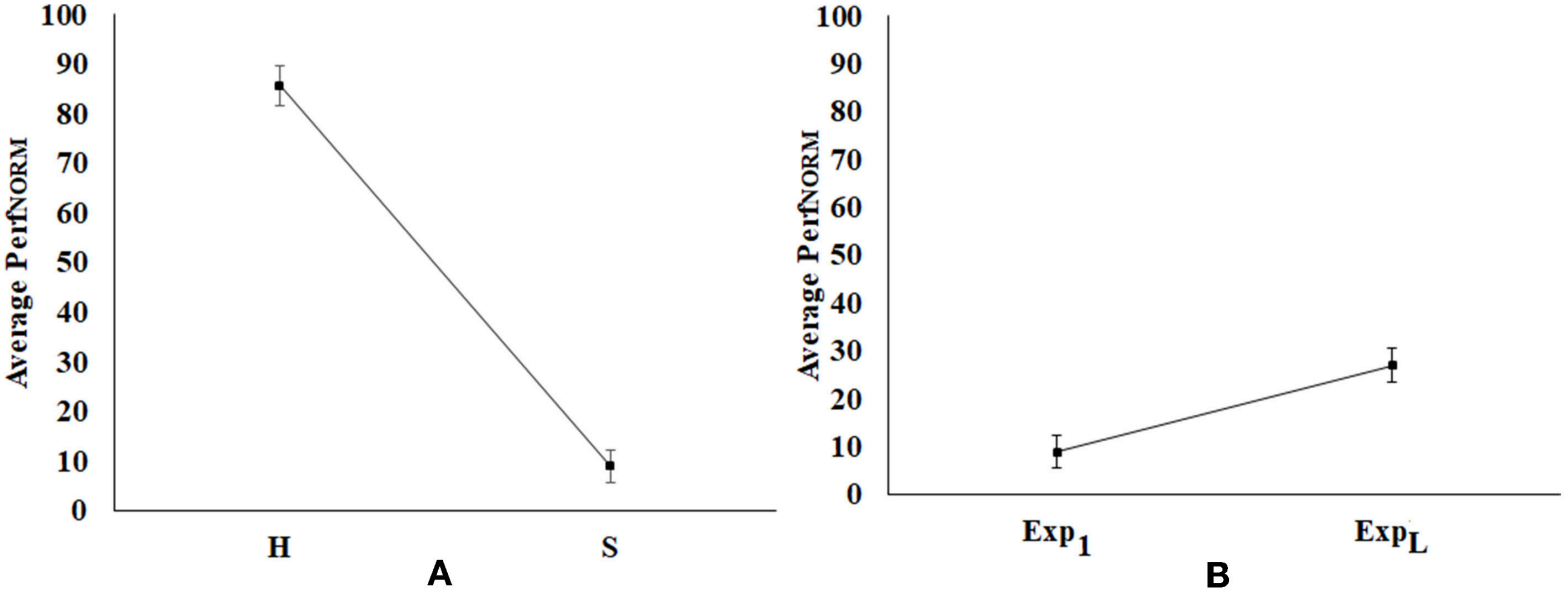
Comparative analysis of normalized performance score **(A)** Healthy (H) vs. Post-stroke (S) Participants, **(B)** Exp_1_ vs. Exp_L_ of Post-stroke Participants. Error bars represent standard error. Exp_1_, First Exposure; Exp_L_, Last Exposure.

While the healthy and post-stroke participants interacted with a varying number of tasks belonging to each difficulty level, we wanted to carry out a comparative analysis between their performances. For the sake of comparison, we computed the *Perf*_*NORM*_ (section Computation of Performance Score) of the healthy and post-stroke participants. Since, the healthy participants had only Exp_1_ in which they achieved Adequate performance in the first task, we carried out a comparative analysis of the *Perf*_*NORM*_ of healthy and post-stroke participants for Exp_1_. Figure 9A indicate the average *Perf*_*NORM*_ (Standard Error bars) of healthy group and post-stroke group. This shows the average *Perf*_*NORM*_ for healthy group is higher than the post-stroke group.

Again, we carried out comparative analysis of *Perf*_*NORM*_ of post-stroke participants during Exp_1_ and Exp_L_ as shown in Figure 9B [average *Perf*_*NORM*_ (Standard Error bars)]. From Figure 9B, we find that multiple exposures to our system has contributed to the overall improvement in functional ability of the post-stroke patients as manifested through the increase in *Perf*_*NORM*_.

Subsequently, we carried out independent sample Wilcoxon rank-sum test on the *Perf*_*NORM*_ of the healthy and post-stroke groups while considering the Exp_1_. Results indicate that these were statistically different (*p*-value < 0.01). Again, we carried out a dependent sample Wilcoxon rank sum test on the *Perf*_*NORM*_ for Exp_1_ and Exp_L_. Results indicate statistical (*p*-value < 0.05) difference in the *Perf*_*NORM*_ between Exp_1_ and Exp_L_.

### Comparative Group Analysis of the Tonic_MEAN_ of Post-stroke Participants During Exp_1_ and Exp_L_ of Stage 2 of the Study

While the post-stroke participants interacted with our performance-sensitive Kinect-assisted HCI task platform, we acquired their event-marked EDA signal (section Physiological Data Acquisition Module) that was processed to extract their *Tonic*_*MEAN*_ corresponding to each task. We wanted to study the implication of Exp_1_ and Exp_L_ on their Tonic activity. For this, we carried out comparative group analysis of the change in Tonic mean from their respective baseline value (Δ*Tonic*_*MEAN*_) (Equation 6) corresponding to tasks of varying difficulty levels between Exp_1_ and Exp_L_. Literature review indicates that the tonic component of the EDA can be associated with a task being executed and the perceived challenge by the post-stroke patient (Chatterjee et al., 2018) with a reduction in tonic activity indicative of one's functional recovery (Clarkson et al., 2010).

**Figure 10:**
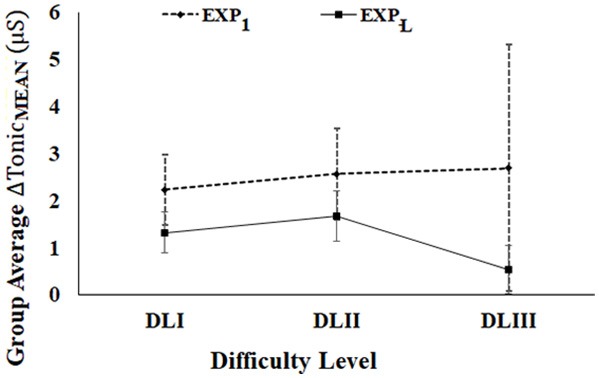
Comparative group analysis of ΔTonic_MEAN_ of stroke participants. Error bars represent standard error.

Figure 10 shows the average Δ*Tonic*_*MEAN*_ (Standard Errors) of post-stroke participants for difficulty levels (DLI to DLIII) over Exp_1_ and Exp_L._ Here, we chose DLI-DLIII for comparative analysis of tonic activation, since none of the post-stroke patients were able to reach DLIV-DLVI tasks during Exp_1_. During the Exp_L_, 100% of the post-stroke participants interacted with tasks in each of DLI, DLII, and DLIII. As can be seen from Figure 10, for each of DLI-DLIII, we see a reduction in the Δ*Tonic*_*MEAN*_ during the Exp_L_ from that during Exp_1_. Such a reduction in their Δ*Tonic*_*MEAN*_ along with their ability to execute tasks with increased challenge with improvement in their task performance score (section Comparative Analysis of Average Performance of Post-stroke Participants in tasks of varying Difficulty Levels during Exp1 and ExpL of Stage 2 of the study) might be indicative of functional recovery (Clarkson et al., 2010) upon repeated exposures.

Subsequently, we carried dependent sample non-parametric Wilcoxon signed rank test on the average Δ*Tonic*_*MEAN*_ over Exp_1_ and Exp_L_ (DLI-DLIII). With the limited number of exposures of the post-stroke participants to our system, the variation in the Δ*Tonic*_*MEAN*_ from Exp_1_ to that during Exp_L_ was not statistically different for the various difficulty levels.

## Discussion

In this work, we have designed a Performance-sensitive Kinect-Assisted HCI task platform for upper limb exercise. The tasks required a participant to stretch out and maneuver his/her hand in physical space while performing a coordinated movement of the upper limb to follow a pattern displayed on the screen of the Task computer. This was accompanied with real-time audio-visual feedback. Subsequently, our system evaluated the task performance that in turn was used to adaptively offer tasks of increasing difficulty in an individualized manner. Also, our system recorded one's physiological signals in a time synchronized manner along with task progression. The overall aim was to understand whether exercising with such a system over multiple exposures can improve functional measures such as reaching and coordination ability in the upper limb of post-stroke survivors in terms of task performance. Additionally, we wanted to understand the implications of exposures on the implicit measures such as physiology of post-stroke patients.

Both healthy and post-stroke participants were exposed to our system. A post-study survey indicated that the tasks by our system were easily understood by both the healthy and post-stroke groups and both the groups appreciated the real-time audio-visual feedback offered by our system. Also, the post-stroke patients expressed their hope that repeated exposure to our system could be beneficial to them toward rehabilitation and mentioned their desire to interact with our system again. While post-stroke patients were offered multiple exposures to our system, we could observe statistically significant improvement in normalized performance score (*Perf*_*NORM*_) from Exp_1_ to Exp_L_. This improved task performance is indicative of an increase in the reaching and coordination ability in their upper limbs. The results of a comparative analysis on their task performance during Exp_1_ and Exp_L_ indicated an improvement in their performance score both within and across difficulty levels. Such an improvement in their task performance score (executing more difficult tasks) coupled with a reduction in Δ*Tonic*_*MEAN*_ might be indicative of their functional recovery (Clarkson et al., 2010) possibly attributed to multiple exposures to our system.

Our present system can serve as a complementary tool in the hands of the therapists involved in stroke rehabilitation. For example, based on the individualized (patient's) capability, the performance-related thresholds used for the switching of tasks can be modulated using a therapist's inputs. Subsequently, the system can be deployed for the patient followed by continuous monitoring of the patient's progress using performance data (stored in backend). In turn, one therapist can cater to the individualized needs of many patients at the same time thereby helping to address the issues of high patient to doctor ratio (in developing countries). Ease of accessibility to such technology-assisted systems can be ensured not only by the availability of such systems at the healthcare centers but also by the cost associated with availing such systems. Our Performance-sensitive Kinect-Assisted HCI task platform can offer a cost-effective solution since it has easy set-up that does not need technical expertise to operate it thereby bringing down the cost of its maintenance. Also, the set-up time required by our system is comparatively less.

Though the results are promising, there are some limitations in terms of participation, device used, assessment approaches, study settings, and follow-up studies that can be addressed in future. The primary limitation is the small sample size of post-stroke participants. In the future, we plan to extend our study incorporating a bigger participant pool. With regard to the device utilized in our study, we have used the Kinect Xbox 360 that was available to us. This has limitations in measurement accuracy that can be attributed to reduced sampling frequency, resolution, etc. (Pfister et al., 2014) compared to the expensive state of the art marker-based techniques (Hingtgen et al., 2006; Perez-Marcos et al., 2017). Again, Kinect Xbox 360 suffers from limitations like relatively poor quality of skeletal tracking (Zhang, 2012) and depth resolution (Pagliari and Livio, 2015) compared to the advanced version such as Kinect 2. However, with the Kinect device being unavailable in the market, we plan to explore the use of other alternatives such as Gesture Xtreme, webcam-based motion capture system, etc. in the future (Knippenberg et al., 2017). Since the execution of tasks offered by our system needed one to stretch his/her hand and make coordinated hand movement by raising the hand against gravity, we expected our post-stroke participants to have at least some limited capacity in their upper extremity. Although we used clinical measure such as FMA scores as one of the inclusion criteria, yet, we did not apply any biomechanical assessment. In the future, we plan to use biomechanical assessment along with clinical measures. For this, we plan to extend our usability study in which we will acquire electromyogram signals from one's upper extremity during execution of such tasks. Thus, added to our preliminary calibration of one's individualized range of motion with the horizontal span of the HCI Task monitor, we plan to perform biomechanical evaluation of the patients similar to that in other studies (Kim et al., 2016; Bakhti et al., 2018; Scano et al., 2018) to tune individualized thresholds. Again, with regard to study settings, our present study offered a limited number of exposures to the post-stroke participants. Also, presently, we have collected data from patients using hospital settings and have not done home-based studies. With respect to the number of exposures, in the future, we plan to design extended studies in which each participant will interact with our system over repeated sessions. Since our system has portability with easy setup options and can easily be deployed in home-based settings, we plan to extend our study to home-based settings in the future. Again, we did not carry out any follow-up study this time. However, we plan to carry out a longitudinal study with subsequent follow-up to investigate whether the patients have been able to translate the skills learnt from the simulated world to real-life settings. The extent of the translation of skills can be quantified in terms of both clinical and functional (ability to execute ADL) measures.

## Conclusion

In this article, we have designed a Performance-sensitive Kinect-Assisted HCI task platform for upper limb exercise and tested its usability with 15 healthy and seven post-stroke participants. The system was reviewed by both healthy and post-stroke participants and the results have indicated the potential of such a system toward upper limb rehabilitation. Additionally, this system can offer a portable, cost-effective, controlled exercise environment for rehabilitation of post-stroke patients. However, open questions still remain on the transferability of the skills learnt from the controlled setting to the real world for quality community living. Nevertheless, our present system can offer a step toward developing a full-fledged exercise platform for individuals suffering from upper limb movement dysfunction.

## Data Availability

The datasets for this study will not be made publicly available because the authors want to state that the research is under process.

## Ethics Statement

The study was carried out in accordance with the recommendations of Institutional Research Ethics, Institutional Ethics Committee (IEC), IIT Gandhinagar. The protocol was approved by the IEC. All the subjects have given the written informed consent in accordance with the declaration of IEC.

## Author Contributions

AD, AY, AC, and UL drafted the manuscript and contributed to the system design, experiment design, experimental data collection with stroke participants, data analysis, and statistical analysis together.

### Conflict of Interest Statement

The authors declare that the research was conducted in the absence of any commercial or financial relationships that could be construed as a potential conflict of interest.

## Footnotes

^1^Microsoft. *Kinect Mouse Cursor – CodePlex Archive*. Available online at: https://archive.codeplex.com/?p=kinectmouse

^2^*Fugl-Meyer Assessment Upper Extremity (FMA-UE) Assessment of Sensorimotor Function*. Available online at: https://neurophys.gu.se/digitalAssets/1520/1520773_fma-ue-protocol-english-updated-20150315.pdf

## References

[B1] Aqueveque P. Ortega P. Pino E. Saavedra F. Germany E. Gómez B. (2017). After stroke movement impairments: a review of current technologies for rehabilitation, in Physical Disabilities-Therapeutic Implications, ed Tan U. (London, UK: InTech), 95–116.

[B2] Bakhti K. K. A. Laffont I. Muthalib M. Froger J. Mottet D. (2018). Kinect-based assessment of proximal arm non-use after a stroke. J. Neuroeng. Rehabil. 15:104. 10.1186/s12984-018-0451-2 30428896 PMC6236999

[B3] Bao X. Mao Y. Lin Q. Qiu Y. Chen S. Li L.. (2013). Mechanism of kinect-based virtual reality training for motor functional recovery of upper limbs after subacute stroke. Neural Regen. Res. 8:2904–2913. 10.3969/j.issn.1673-5374.2013.31.003 25206611 PMC4146174

[B4] Boettger S. Christian P. Yeragani V. K. Donath L. Mueller H- J. Holger H. W.. (2010). Heart rate variability, QT variability, and electrodermal activity during exercise. Med. Sci. Sports Exercise 42, 443–448. 10.1249/MSS.0b013e3181b64db1 19952826

[B5] Borghese A. Michele P. Renato M. Lanzi P. L. (2012). An integrated low-cost system for at-home rehabilitation, in 2012 18th International Conference on Virtual Systems and Multimedia (Milan: IEEE), 553–556.

[B6] Carr J. H. Shepherd R. B. Nordholm L. Lynne D. (1985). Investigation of a new motor assessment scale for stroke patients. Phys. Ther. 65, 175–180. 10.1093/ptj/65.2.175 3969398

[B7] Chang C. Y. Lange B. Zhang M. Koenig S. Requejo P. Somboon N. (2012). Towards pervasive physical rehabilitation using Microsoft Kinect, in The 6th International Conference on Pervasive Computing Technologies for Healthcare and Workshops, 2012 (San Diego, CA), 159–162.

[B8] Chatterjee S. Daly J. J. Porges E. C. Fox E. J. Rose D. K. McGuirk T. E.. (2018). Mobility function and recovery after stroke: preliminary insights from sympathetic nervous system activity. J. Neurol. Phys. Ther. 42, 224–232. 10.1097/NPT.0000000000000238 30138228 PMC6156783

[B9] Clarke D. J. (2014). Nursing practice in stroke rehabilitation: systematic review and meta-ethnography. J. Clin. Nurs. 23, 1201–1226. 10.1111/jocn.12334 24102924

[B10] Clarkson A. N. Huang B. S. MacIsaac S. E. Mody I. Carmichael S. T. (2010). Reducing excessive GABA-mediated tonic inhibition promotes functional recovery after stroke. Nature 468, 305–309. 10.1038/nature09511 21048709 PMC3058798

[B11] Deo M. G. (2013). Doctor population ratio for India-the reality. Indian J. Med. Res. 137, 632–635. 23703329 PMC3724242

[B12] Gaddis G. M. Gaddis M. L. (1990). Introduction to biostatistics: part 5, statistical inference techniques for hypothesis testing with nonparametric data. Ann. Emergency Med. 19, 1054–1059. 10.1016/S0196-0644(05)82571-5 2203291

[B13] Gauthier L. V. Chelsea K. Alexandra B. Nancy S. Gitendra U. Edward T.. (2017). Video game rehabilitation for outpatient stroke (VIGoROUS): protocol for a multi-center comparative effectiveness trial of in-home gamified constraint-induced movement therapy for rehabilitation of chronic upper extremity hemiparesis. BMC Neurol. 17:109. 10.1186/s12883-017-0888-0 28595611 PMC5465449

[B14] George S. H. Rafiei M. H. Borstad A. Adeli H. Gauthier L. V. (2017). Gross motor ability predicts response to upper extremity rehabilitation in chronic stroke. Behav. Brain Res. 333, 314–322. 10.1016/j.bbr.2017.07.002 28688897 PMC5583064

[B15] Gil-Gómez J. A. Manzano-Hernández P. Albiol-Pérez S. Aula-Valero C. Gil-Gómez H. Lozano-Quilis J. A. (2017). USEQ: a short questionnaire for satisfaction evaluation of virtual rehabilitation systems. Sensors 17:1589. 10.3390/s17071589 28686174 PMC5539644

[B16] Green T. L. King K. M. (2010). Functional and psychosocial outcomes 1 year after mild stroke. J. Stroke Cerebrovasc. Dis. 19, 10–16. 10.1016/j.jstrokecerebrovasdis.2009.02.005 20123221

[B17] Hidler J. Nichols D. Pelliccio M. Brady K. (2005). Advances in the understanding and treatment of stroke impairment using robotic devices. Topics Stroke Rehabil. 12, 22–35. 10.1310/RYT5-62N4-CTVX-8JTE 15940582

[B18] Hingtgen B. McGuire J. R. Wang M. Harris G. F. (2006). An upper extremity kinematic model for evaluation of hemiparetic stroke. J. Biomechan. 39, 681–688. 10.1016/j.jbiomech.2005.01.008 16439237

[B19] Huber M. E. Seitz A. L. Leeser M. Sternad D. (2015). Validity and reliability of Kinect skeleton for measuring shoulder joint angles: a feasibility study. Physiotherapy 101, 389–393. 10.1016/j.physio.2015.02.002 26050135 PMC4600010

[B20] Jack K. McLean S. M. Moffett J. K. Gardiner E. (2010). Barriers to treatment adherence in physiotherapy outpatient clinics: a systematic review. Manual Ther. 15, 220–228. 10.1016/j.math.2009.12.004 20163979 PMC2923776

[B21] Khoshelham K. Elberink S. O. (2012). Accuracy and resolution of kinect depth data for indoor mapping applications. Sensors 12, 1437–1454. 10.3390/s120201437 22438718 PMC3304120

[B22] Kim W- S Sungmin C. Dongyoub B. Hyunwoo B. Nam-Jong P. (2016). Upper extremity functional evaluation by Fugl-Meyer assessment scoring using depth-sensing camera in hemiplegic stroke patients. PLoS ONE 11:e0158640. 10.1371/journal.pone.0158640 27367518 PMC4930182

[B23] Knippenberg E. Jonas V. Ilse L. Steven P. Annick T. Annemie S. (2017). Markerless motion capture systems as training device in neurological rehabilitation: a systematic review of their use, application, target population and efficacy. J. Neuroeng. Rehabil. 14:61. 10.1186/s12984-017-0270-x 28646914 PMC5482943

[B24] Krebs H. I. Ferraro M. Buerger S. P. Newbery M. J. Makiyama A. Sandmann M.. (2004). Rehabilitation robotics: pilot trial of a spatial extension for MIT-Manus. J. NeuroEng. Rehabil. 1:5. 10.1186/1743-0003-1-5 15679916 PMC544952

[B25] Lange B. Chang C. Y. Suma E. Newman B. Rizzo A. S. Bolas M. (2011). Development and evaluation of low cost game-based balance rehabilitation tool using the Microsoft Kinect sensor, in Engineering in Medicine and Biology Society, EMBC, 2011 Annual International Conference of the IEEE (Boston, MA: IEEE), 1831–1834. 10.1109/IEMBS.2011.6090521 22254685

[B26] Liao W. W. McCombe Waller S. Whitall J. (2018). Kinect-based individualized upper extremity rehabilitation is effective and feasible for individuals with stroke using a transition from clinic to home protocol. Cogent Med. 5:1428038 10.1080/2331205X.2018.1428038

[B27] Liu L. Chen X. Lu Z. Cao S. Wu D. Zhang X. (2017). Development of an EMG-ACC-based upper limb rehabilitation training system. IEEE Transac. Neural Syst. Rehabil. Eng. 25, 244–253. 10.1109/TNSRE.2016.2560906 28113559

[B28] MacIntosh B. J. McIlroy W. E. Mraz R. Staines W. R. Black S. E. Graham S. J. (2008). Electrodermal recording and fMRI to inform sensorimotor recovery in stroke patients. Neurorehabil. Neural Repair 22, 728–736. 10.1177/1545968308316386 18784267 PMC4896813

[B29] Magermans D. J. Chadwick E. K. Veeger H. E. van der Helm F. C. (2005). Requirements for upper extremity motions during activities of daily living. Clin. Biomech. 20, 591–599. 10.1016/j.clinbiomech.2005.02.006 15890439

[B30] Masia L. Casadio M. Giannoni P. Sandini G. Morasso P. (2009). Performance adaptive training control strategy for recovering wrist movements in stroke patients: a preliminary, feasibility study. J. Neuroeng. Rehabil. 6:44. 10.1186/1743-0003-6-44 19968873 PMC2799433

[B31] Metzger J. C. Lambercy O. Califfi A. Dinacci D. Petrillo C. Rossi P.. (2014). Assessment-driven selection and adaptation of exercise difficulty in robot-assisted therapy: a pilot study with a hand rehabilitation robot. J. Neuroeng. Rehabil. 11:154. 10.1186/1743-0003-11-154 25399249 PMC4273449

[B32] Oldfield R. C. (1971). The assessment and analysis of handedness: the Edinburgh inventory. Neuropsychologia 9, 97–113. 10.1016/0028-3932(71)90067-4 5146491

[B33] Pagliari D. Livio P. (2015). Calibration of kinect for xbox one and comparison between the two generations of microsoft sensors. Sensors 15, 27569–27589. 10.3390/s151127569 26528979 PMC4701245

[B34] Pastor I. Hayes H. A. Bamberg S. J. (2012). A feasibility study of an upper limb rehabilitation system using kinect and computer games, in 2012 Annual International Conference of the IEEE Engineering in Medicine and Biology Society, (San Diego, CA: IEEE), 1286–1289. 10.1109/EMBC.2012.6346173 23366134

[B35] Perez-Marcos D. Chevalley O. Schmidlin T. Garipelli G. Serino A. Vuadens P.. (2017). Increasing upper limb training intensity in chronic stroke using embodied virtual reality: a pilot study. J. Neuroeng. Rehabil. 14:119. 10.1186/s12984-017-0328-9 29149855 PMC5693522

[B36] Pfister A. West A. M. Bronner S. Noah J. A. (2014). Comparative abilities of microsoft kinect and vicon 3D motion capture for gait analysis. J. Med. Eng. Technol. 38, 274–280. 10.3109/03091902.2014.909540 24878252

[B37] Roy A. K. Soni Y. Dubey S. (2013). Enhancing effectiveness of motor rehabilitation using kinect motion sensing technology, in Global Humanitarian Technology Conference: South Asia Satellite (GHTC-SAS), 2013 IEEE (Trivandrum: IEEE), 298–304.

[B38] Scano A. Andrea C. Tosatti L. M. Müller M. Atzori M. (2018). Muscle synergy analysis of a hand-grasp dataset: a limited subset of motor modules may underlie a large variety of grasps. Front. Neurorobot. 12:57. 10.3389/fnbot.2018.00057 30319387 PMC6167452

[B39] Sin H. Lee G. (2013). Additional virtual reality training using Xbox Kinect in stroke survivors with hemiplegia. Am. J. Phys. Med. Rehabil. 92, 871–880. 10.1097/PHM.0b013e3182a38e40 24051993

[B40] Singer B. Garcia-Vega J. (2017). The Fugl-Meyer upper extremity scale. J. Physiother. 63, 53. 10.1016/j.jphys.2016.08.010 27964964

[B41] Squeri V. Masia L. Giannoni P. Sandini G. Morasso P. (2014). Wrist rehabilitation in chronic stroke patients by means of adaptive, progressive robot-aided therapy. IEEE Trans. Neural Syst. Rehabil. Eng. 22, 312–325. 10.1109/TNSRE.2013.2250521 23508271

[B42] Sucar L. E. Luis R. Leder R. Hernández J. Sánchez I. (2010). Gesture therapy: a vision-based system for upper extremity stroke rehabilitation, in Engineering in Medicine and Biology Society (EMBC), 2010 Annual International Conference of the IEEE (Buenos Aires: IEEE), 3690–3693. 10.1109/IEMBS.2010.5627458 21096856

[B43] Tao G. Archambault P. S. Levin M. F. (2013). Evaluation of kinect skeletal tracking in a virtual reality rehabilitation system for upper limb hemiparesis, in 2013 International Conference on Virtual Rehabilitation (ICVR) (IEEE), 164–165.

[B44] Turolla A. Dam M. Ventura L. Tonin P. Agostini M. Zucconi C.. (2013). Virtual reality for the rehabilitation of the upper limb motor function after stroke: a prospective controlled trial. J. Neuroeng. Rehabil. 10:85. 10.1186/1743-0003-10-85 23914733 PMC3734026

[B45] Vieyra C. Vieyra R. (2018). Physics Toolbox Sensor Suite Application, google.play.store. Available online at: http://www.strokecenter.org/patients/about-stroke/stroke-statistics/1

[B46] Wang F. Barkana D. E. Sarkar N. (2013). Development of a versatile robotic system with multiple training modes for upper-limb rehabilitation study. Adv. Robot. Autom. 2:112 10.4172/2168-9695.1000112

[B47] Webster D. Ozkan C. (2014). Systematic review of Kinect applications in elderly care and stroke rehabilitation. J. Neuroeng. Rehabil. 11:108. 10.1186/1743-0003-11-108 24996956 PMC4094409

[B48] Yang C. Kerr A. Stankovic V. Stankovic L. Rowe P. (2014). Upper limb movement analysis via marker tracking with a single-camera system, in 2014 IEEE International Conference on Image Processing (ICIP) (Paris: IEEE), 2285–2289.

[B49] Young B. M. Nigogosyan Z. Remsik A. Walton L. M. Song J. Nair V. A.. (2014). Changes in functional connectivity correlate with behavioral gains in stroke patients after therapy using a brain-computer interface device. Front. Neuroeng. 7:25. 10.3389/fneng.2014.00025 25071547 PMC4086321

[B50] Zhang Z. (2012). Microsoft kinect sensor and its effect. IEEE Multimedia 19, 4–10. 10.1109/MMUL.2012.24

